# Oral Anticoagulants in Patients with Atrial Fibrillation and Active Cancer

**DOI:** 10.31083/j.rcm2307242

**Published:** 2022-06-27

**Authors:** Li-Ying Yu, Yen-Wen Liu, Tzu-Yu Chou, Yi-Chia Liu, Pei-Fang Su, Ping-Yen Liu

**Affiliations:** ^1^Division of Cardiology, Department of Internal Medicine, National Cheng Kung University Hospital, College of Medicine, National Cheng Kung University, 704 Tainan, Taiwan; ^2^Institute of Clinical Medicine, College of Medicine, National Cheng Kung University, 704 Tainan, Taiwan; ^3^The Center for Quantitative Sciences, Clinical Medicine Research Center, National Cheng Kung University Hospital, 704 Tainan, Taiwan; ^4^Department of Statistics, National Cheng Kung University, 701 Tainan, Taiwan

**Keywords:** cancer, atrial fibrillation, oral anticoagulants

## Abstract

**Background:**

Atrial fibrillation (AF) is associated with an increased risk of heart failure, death and thromboembolism. AF is prevalent in patients with cancer. Although current guidelines suggest the application of oral anticoagulants (OACs) for thromboembolic event prevention in high-risk AF patients, owing to the high thromboembolic and bleeding risks of active-cancer patients, there is no consensus on the use of OACs in such a population. Therefore, we conducted this retrospective cohort study to investigate the applicability of the ${\mathrm{CHA}}_{2}$${\mathrm{DS}}_{2}$-VASc score and to evaluate the efficacy and safety outcomes of OAC therapy in active-cancer patients with AF.

**Methods:**

This retrospective cohort study enrolled patients diagnosed with cancer at National Cheng Kung University Hospital between November 2012 and August 2019. The primary outcomes included all-cause mortality, thromboembolic events (stroke/transient ischemic attack and systemic emboli), acute myocardial infarction (AMI), hospitalization for HF and major bleeding events.

**Results:**

We enrolled 2429 patients with active cancer. Among these patients, 1060 patients (43.6%) had AF. After 1:2 propensity score matching, 690 cancer patients with AF were enrolled for the final analysis, grouped as follows: 225 patients taking OACs and 465 patients without OAC treatment. The OAC-treated group had lower all-cause mortality than the patients without OAC treatment (all-cause mortality rate in OAC treatment *vs*. non-OAC treatment: 24.4% *vs*. 37.4%, hazard ratio 0.58 [95% confidence interval (CI) 0.43–0.78], *p *< 0.001). However, there was no difference in thromboembolic events, myocardial infarction or heart failure hospitalization between the OAC-treated and non-OAC-treated groups. Importantly, the risk of major bleeding composition (i.e., major gastrointestinal bleeding and intracranial hemorrhage) was similar between these two groups. Moreover, the ${\mathrm{CHA}}_{2}$${\mathrm{DS}}_{2}$-VASc score could not predict thromboembolic events in the enrolled active-cancer patients with AF (OR 1.23, 95% CI 0.98–1.56).

**Conclusions:**

OAC treatment may significantly reduce the risk of death, without safety concerns, in active-cancer patients with AF. OAC treatment may not prevent thromboembolic events in patients with active cancer and AF. However, we found that OAC treatment is associated with improved prognosis without increasing the risks of major bleeding, despite several limitations in this study. Further studies are required to determine the optimal use of anticoagulation therapy in this high-risk population.

## 1. Introduction

Atrial fibrillation (AF) is the most common type of cardiac arrhythmia worldwide. It causes a significant burden to patients, physicians, and the health care system owing to its high morbidity and mortality, including heart failure, death, thromboembolism (e.g., ischemic stroke and systemic emboli), and subsequent anticoagulation-related bleeding events [1, 2, 3, 4]. AF is prevalent in patients with cancer and may be present during anticancer treatment, at the time of diagnosis, or even a period of time after cancer therapy [1]. However, the mechanisms underlying the association between cancer and AF remain unclear [5].

Cancers are associated with an increased risk of thromboembolism due to multiple risk factors, such as hypercoagulability, overproduction of inflammatory cytokines, and compression or invasion of blood vessels [6, 7]. However, patients with active cancers are also at higher risk of bleeding, possibly due to endothelial dysfunction, thrombocytopenia, or thrombocyte dysfunction, especially during anticancer therapies [5]. These opposing characteristics challenge the clinician on how to best manage the hypercoagulable state without causing major bleeding.

Currently, there is no consensus on the use of oral anticoagulants (OACs) to prevent thromboembolism in patients with active cancer and AF. Additionally, it is indicated that the popular AF risk-stratification models, namely, the CHA₂DS₂-VASc and HAS-BLED scores, may not be appropriate for cancer patients with AF to predict the risk of AF-related thromboembolism and OAC-related major bleeding events, respectively [8, 9]. Therefore, this retrospective cohort study was conducted to verify the applicability of the ${\mathrm{CHA}}_{2}$${\mathrm{DS}}_{2}$-VASc score and to assess the efficacy and safety outcomes of OAC therapy in active-cancer patients with AF.

## 2. Patients and Methods

### 2.1 Study Subjects

In this retrospective cohort study, patients with active cancer were recruited at the National Cheng Kung University Hospital from November 2012 to August 2019. The cancer diagnosis of the enrolled patients was based on the medical records of the International Classification of Diseases-9 codes, including liver, colorectal, lung, urologic (kidney, renal pelvis, ureter, and urinary bladder), breast, prostate, upper gastrointestinal (esophageal, gastric, duodenal), biliary tract, pancreatic, and gynecologic (vaginal, vulvar, cervical, and uterine) cancers, hematologic disorder (leukemia and lymphoma), brain tumor, skin, oral, and nasopharyngeal carcinoma. Clinical information, including physiological data and underlying comorbidities, was collected from electronic medical records on the date of enrollment. Demographic characteristics included age, sex, body weight, body height, body mass index, diabetes mellitus, hypertension, dyslipidemia, coronary artery disease, myocardial infarction, heart failure (HF), hypertrophic cardiomyopathy (HCM), stroke, peripheral artery disease, deep vein thrombosis, and chronic kidney disease.

**Fig. 1.**
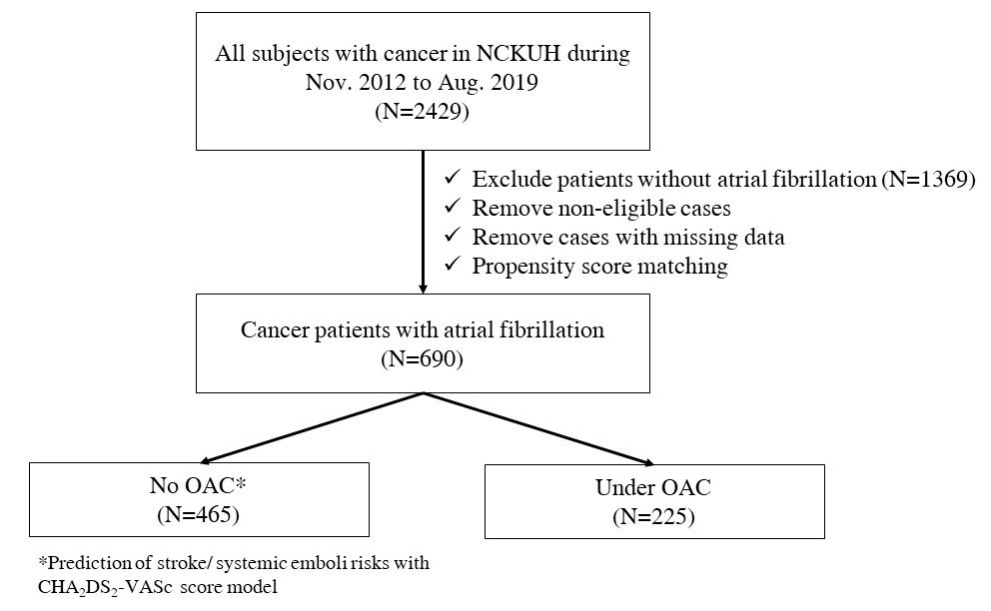
**Flowchart of the study design and processes**.

Patients who met all the following criteria were included: (1) age ≥20 years; (2) diagnosis of active cancer and AF; and (3) follow-up for at least three months or longer after the diagnosis of cancer. Patients were excluded if one of the following criteria were met: (1) end-stage renal disease on dialysis; (2) severe hepatic disease; and (3) high-risk thrombophilic conditions, such as antiphospholipid syndrome and severe thrombocytopenia (platelet count <20,000/μL). All enrolled patients were followed-up until July 2021 or until the date of death. The prescriptions for oral anticoagulants (OACs), including direct oral anticoagulants (dabigatran, rivaroxaban, apixaban, and edoxaban) and vitamin K antagonists, were also recorded. After propensity score matching, cancer patients with AF were divided into OAC and non-OAC groups (Fig. 1).

### 2.2 Clinical Outcomes

#### 2.2.1 Effectiveness Endpoints

The primary effectiveness endpoints were all-cause mortality and the composition of stroke/transient ischemic attack (TIA) and systemic emboli (SE). Acute myocardial infarction (AMI), hospitalization for HF, stroke/TIA, and SE were defined as secondary effectiveness endpoints.

#### 2.2.2 Safety Endpoints

Based on the criteria of the International Society on Thrombosis and Hemostasis, the safety outcome was the composite of major bleeding events, including (1) clinically overt gastrointestinal (GI) bleeding accompanied by a decrease in the hemoglobin level of at least 2 g/dL or transfusion of at least 2 units of packed red blood cells, (2) occurrence of intracranial hemorrhage (ICH), or (3) death.

### 2.3 Statistical Analysis

Numeric and dichotomous data are presented as the mean ± standard deviation and numbers (percentages), respectively. Demographic and clinical baseline characteristics between the AF and active-cancer patients with and without OAC were compared using the Pearson Chi-square test or Fisher’s exact test and Student’s *t* test for categorical variables and continuous variables, respectively. We used propensity score matching to construct a maximally randomized model, especially concerning factors known to contribute to thromboembolic events among the general population. When more than one endpoint occurred within the follow-up period, only the first event was used for analysis. The cumulative events of clinical outcomes were assessed using the Cox proportional hazards model. To control for confounding factors, a multivariate Cox regression model was performed. Univariate Cox regression analysis was performed to evaluate factors related to the primary effectiveness and safety endpoints, while factors with *p *< 0.1 on univariate analysis were considered in the multivariate Cox regression analysis to estimate the adjusted hazard ratio (HR) with 95% confidence intervals (CI). Differences were considered statistically significant at *p *< 0.05. All statistical analyses were performed using SPSS software (Version 24.0, SPSS Inc., Chicago, IL, USA).

## 3. Results

**Fig. 2.**
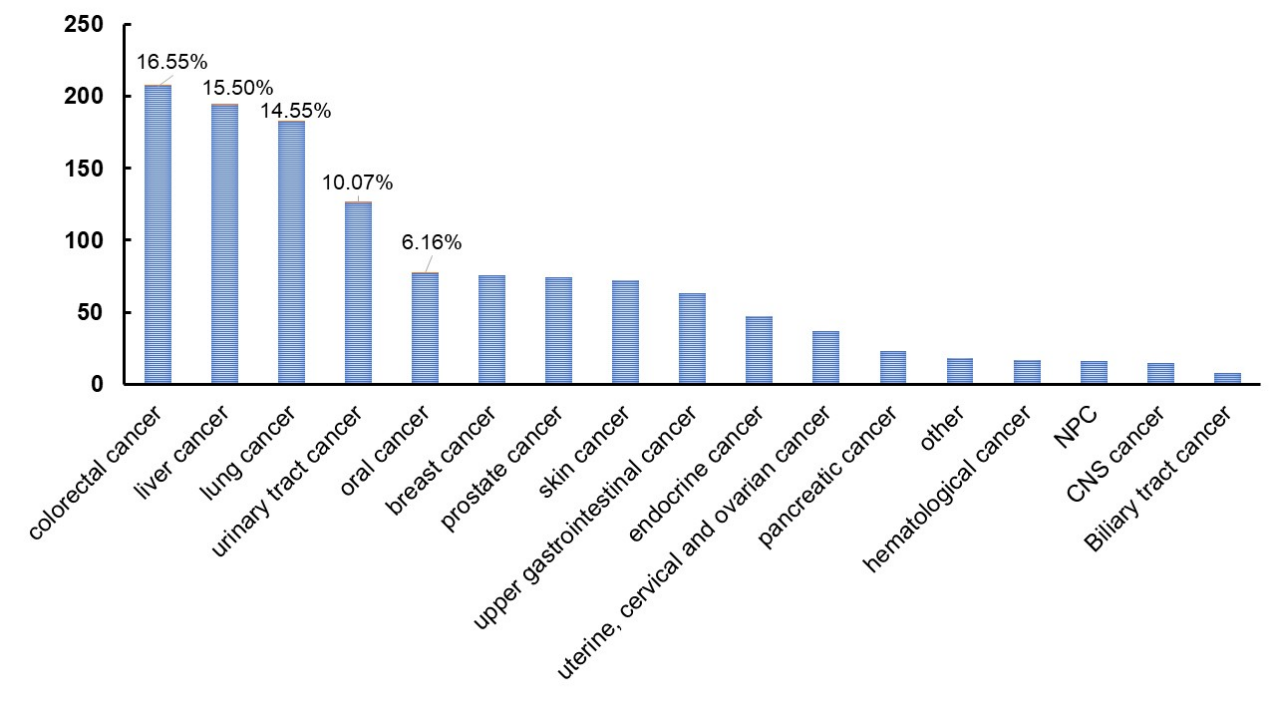
**Distribution of cancer diagnoses**.

A total of 2429 patients with active cancer were enrolled in this study. There were 1060 patients (age, 75.2 ± 10.6 years; female, 40.8%) with AF (Table 1). Only 326 cancer patients (30.8%) with AF received OACs. The baseline characteristics of this cohort are summarized in Table 1. However, there were several significantly different variables between patients with and without OAC; therefore, propensity score matching was conducted for further analysis. Ultimately, 690 cancer patients with AF were enrolled in the analysis, comprising 225 patients taking OACs and 465 patients without OAC treatment. The top five cancer diagnoses were colorectal cancer (17%), hepatocellular cancer (15%), lung cancer (15%), genitourinary cancer (10%), and oral cancer (6%) (Table 2 and Fig. 2).

**Table 1.** **Baseline characteristics of cancer patients with atrial fibrillation before and after propensity score matching**.

Characteristics	Before propensity score matching				After propensity score matching			
	Overall N = 1060	OAC		*p*	Overall N = 690	OAC		*p*
		Yes N = 326	No N = 734			Yes N = 225	No N = 465	
Age	75.16 (10.55)	74.08 (9.80)	75.64 (10.84)	0.007	74.94 (10.81)	74.74 (9.86)	75.04 (11.24)	0.44
Male (%)	627 (59.2%)	187 (57.4%)	440 (59.9%)	0.43	414 (60.0%)	121 (53.8%)	293 (63.0%)	0.02
Diabetes mellitus (%)	401 (37.8%)	131 (40.2%)	270 (36.8%)	0.29	284 (41.2%)	84 (37.3%)	200 (43.0%)	0.16
Dyslipidemia (%)	561 (52.9%)	219 (67.2%)	342 (46.6%)	<0.001	412 (59.7%)	149 (66.2%)	263 (56.6%)	0.02
Hypertension (%)	807 (76.1%)	543 (74.0%)	264 (81.0%)	0.01	566 (82.0%)	193 (85.8%)	373 (80.2%)	0.07
Stroke (%)	199 (18.8%)	72 (22.1%)	127 (17.3%)	0.07	146 (21.2%)	54 (24.0%)	92 (19.8%)	0.20
Coronary artery disease (%)	239 (22.5%)	84 (25.8%)	155 (21.1%)	0.10	185 (26.8%)	60 (26.7%)	125 (26.9%)	0.95
Chronic kidney disease (%)	388 (36.6%)	105 (32.2%)	283 (38.6%)	0.048	245 (35.5%)	77 (34.2%)	168 (36.1%)	0.62
Myocardial infarction (%)	74 (7.0%)	24 (7.4%)	50 (6.8%)	0.75	59 (8.6%)	19 (8.4%)	40 (8.6%)	0.95
Heart failure (%)	349 (32.9%)	142 (43.6%)	207 (28.2%)	<0.001	262 (38.0%)	97 (43.1%)	165 (35.5%)	0.053
Peripheral artery disease (%)	51 (4.8%)	24 (7.4%)	27 (3.7%)	0.01	35 (5.1%)	14 (6.2%)	21 (4.5%)	0.34
Deep vein thrombosis (%)	21 (2.0%)	2 (0.6%)	19 (2.6%)	0.03	12 (1.7%)	2 (0.9%)	10 (2.2%)	0.36
${\mathrm{CHA}}_{2}$${\mathrm{DS}}_{2}$­-VASc score	3.93 (1.83)	4.15 (1.74)	3.83 (1.86)	0.005	4.15 (1.81)	4.30 (1.66)	4.07 (1.88)	0.14
${\mathrm{CHA}}_{2}$${\mathrm{DS}}_{2}$­-VASc 0 or 1	94 (8.9%)	20 (6.1%)	74 (10.0%)	**-**	50 (7.2%)	10 (4.4%)	40 (8.6%)	0.06
Aspirin (%)	161 (15.2%)	44 (13.5%)	117 (15.9%)	0.31	109 (15.8%)	32 (14.2%)	77 (16.6%)	0.43
P2Y12 inhibitors (%)	120 (11.3%)	44 (13.5%)	76 (10.3%)	0.20	99 (14.1%)	32 (14.2%)	67 (14.4%)	0.76
ACEI/ARB	227 (21.4%)	128 (39.3%)	99 (13.5%)	<0.001	161 (23.3%)	74 (32.9%)	87 (18.7%)	<0.001
statin	215 (20.3%)	108 (33.1%)	107 (14.6%)	<0.001	162 (23.5%)	71 (31.6%)	91 (19.6%)	<0.001

ACEI/ARB, Angiotensin converting enzyme inhibitor/Angiotensin II receptor blockers; OAC, oral anticoagulant.

**Table 2.** **Cancer diagnosis of the enrolled patients after propensity score matching**.

Cancer diagnosis	Prevalence
Colorectal cancer	17%
Hepatocellular cancer	15%
Lung cancer	15%
Genitourinary cancer	10%
Oral cancer	6%
Others	37%

The median follow-up duration was 3.2 (interquartile range, 1.6–5.1) years. Both groups had high ${\mathrm{CHA}}_{2}$${\mathrm{DS}}_{2}$-VASc scores. Compared to cancer-and-AF patients without OAC treatment, OAC-treated cancer-and-AF patients had a better prognosis, with lower all-cause mortality (OAC-treated *vs*. no OAC: 24.4% *vs*. 37.4%, HR 1.84 [95% CI 0.43–0.78], *p *< 0.001, Table 3). However, the incidence rate of stroke/TIA and SE between the OAC-treated group and no OAC group was not different (OAC-treated *vs*. no OAC: 8.4% *vs*. 4.7%, HR 0.58 [95% CI 0.99–3.4], *p* = 0.06, Table 2). Additionally, the difference in the incidence of either AMI or HF hospitalization between the 2 groups (Table 3) was not significant, either.

**Table 3.** **Association between OAC use and primary endpoints in patients with cancer and atrial fibrillation**.

Variables		OAC		Hazard ratio (95% CI)	*p*
		Yes	No		
Effectiveness endpoints					
	Stroke/SE	19 (8.4%)	22 (4.7%)	1.84 (0.99, 3.40)	0.06
	Death	55 (24.4%)	174 (37.4%)	0.58 (0.43, 0.78)	<0.001
	AMI	7 (3.1%)	24 (5.2%)	0.60 (0.26, 1.40)	0.24
	HF hospitalization	14 (6.2%)	27 (5.8%)	1.09 (0.57, 2.08)	0.79
Safety endpoints					
	Composition of major bleeding	8 (3.6%)	20 (4.3%)	0.82 (0.34, 1.83)	0.64
	Major GI bleeding	4 (1.8%)	16 (3.4%)	0.51 (0.14, 1.4)	0.23
	ICH	1 (0.4%)	2 (0.4%)	1.03 (0.05, 10.84)	0.98

AMI, acute myocardial infarction; GI bleeding, gastrointestinal bleeding; HF, heart failure; ICH, intracranial hemorrhage; OAC, oral anticoagulant; SE, systemic embolism.

The risk of major bleeding composition (major GI bleeding and ICH) was similar between the two groups (OAC-treated *vs*. no OAC: 3.6% *vs*. 4.3%, HR 0.82 [95% CI 0.34–1.83], *p* = 0.64, Table 3). Neither major GI bleeding nor ICH risk was significantly different between the two groups (Table 3).

Finally, stroke/TIA and SE events were identified in the nontreated group. The ${\mathrm{CHA}}_{2}$${\mathrm{DS}}_{2}$-VASc score was calculated for all patients with cancer and for AF patients without OAC treatment. Stroke/TIA and SE were most prevalent among patients with ${\mathrm{CHA}}_{2}$${\mathrm{DS}}_{2}$-VASc scores of 3 to 5. The risks did not increase as the scores increased (Table 4).

**Table 4.** **${\mathrm{CHA}}_{2}$${\mathrm{DS}}_{2}$-VASc score distribution of all subjects**.

${\mathrm{CHA}}_{2}$${\mathrm{DS}}_{2}$-VASc score	OAC (+), n = 225		OAC (-), n = 465	
	Stroke/SE, n (%)	Total (%)	Stroke/SE, n (%)	Total (%)
0	1 (33.3%)	3 (1%)	0 (0%)	17 (4%)
1	0 (0%)	7 (3%)	1 (4.3%)	23 (5%)
2	1 (6.3%)	16 (7%)	0 (0%)	51 (11%)
3	1 (2.2%)	46 (20%)	5 (6.1%)	82 (18%)
4	5 (9.4%)	53 (24%)	4 (3.7%)	108 (23%)
5	5 (9.8%)	51 (23%)	5 (6.0%)	83 (18%)
6	3 (10%)	30 (13%)	2 (4.1%)	49 (11%)
7	3 (27.3%)	11 (5%)	4 (10.5%)	38 (8%)
8	0 (0%)	6 (3%)	1 (10%)	10 (2%)
9	0 (0%)	2 (1%)	0 (0%)	4 (1%)

OAC, oral anticoagulant; SE, systemic embolism.

## 4. Discussion

This study revealed that compared to the active cancer and AF patients without taking OACs, those active cancer and AF patients with OAC treatment did not have lower risk of stroke/TIA and SE. However, these OAC-treated cancer patients with AF had lower all-cause mortality rate. Furthermore, OAC treatment did not increase the risk of major bleeding, such as major gastrointestinal bleeding and ICH.

Given improvements in cancer treatment, the survival rate of cancer patients has increased. The coexistence of cancer and AF is becoming increasingly prevalent, and clinicians are likely to encounter an increasing number of patients with comorbid conditions. The increased risk of thromboembolism and bleeding challenges clinicians in deciding whether to initiate anticoagulation and how to choose an anticoagulant. Nevertheless, there are no guidelines or recommendations for the treatment of this high-risk population. The 2020 European Society of Cardiology guidelines for AF recommended a multidisciplinary team to make decisions regarding thromboprophylaxis in cancer patients because these patients may have multiple comorbidities, such as renal failure, hepatic failure, thrombocytopenia, obesity, or cachexia, and drug–drug interactions between OACs and cancer therapy regimens [4]. Several observational studies investigated the efficacy and safety of direct oral anticoagulants (DOACs) and vitamin K antagonist (warfarin) in patients with active cancer and AF and showed the efficacy and safety of OACs [10, 11, 12, 13]. Major trials regarding stroke prevention for patients with atrial fibrillation carried subgroup analyses. In ARISTOLTE, ROCKET AF, and ENGAGE AF-TIMI 48 trials, the relative efficacy and safety of DOACs compared with warfarin were not significantly different in patients with and without active cancer [10, 11, 14]. However, current existing data have focused on efficacy and safety in the comparison of different OAC treatments in patients with active cancer and AF [11]. The efficacy and safety of using OAC treatment are left to be explored. Moreover, the prescription of OACs is often hindered by the fear of bleeding in our current practice.

Regarding bleeding tendency, the OAC subgroup did not show a significant increase in the risk of GI bleeding and ICH compared to that in AF cancer patients without OAC. The results may be associated with a lack of data to stratify indications and dosage of OACs, site(s) or complexity of cancer, and most importantly, a previous history of bleeding events. The OAC subgroup may have been in better condition, regardless of the comorbidities.

### Limitations

This study has several limitations. First, the study population was limited after propensity score matching. Second, the cancer population enrolled in the study was based on ICD-9 coding because these codes reflect only the sites of cancer. There was no information regarding the timing of cancer diagnosis, the stage of cancer, adoption of anticancer therapies, or therapeutic response in these patients. Moreover, the clinical conditions and indications at the time when OACs were prescribed were not known. Third, the data of index events, including death, stroke/SE, AMI, HF hospitalization, GI bleeding, and ICH, were obtained from the electronic medical records of a single hospital. Medical records from other hospitals were not available. Fourth, AF, particularly paroxysmal AF, may be underdiagnosed. Finally, the possibility of selection bias and incomplete patient records cannot be excluded. Additional prospective studies are warranted to confirm the results of the present study.

## 5. Conclusions

OAC treatment may significantly reduce the risk of death, without safety concerns, in active-cancer patients with AF. Further studies are required to determine the optimal use of anticoagulation therapy in this high-risk population.
